# IRIS associated with tuberculosis of CNS in HIV and non-HIV infected patients: how long do we need to use steroids

**DOI:** 10.1186/1471-2334-14-S7-P42

**Published:** 2014-10-15

**Authors:** Adriana Hristea, Daniela Munteanu, Raluca Jipa, Raluca Mihăilescu, Eliza Manea, Raluca Hrişcă, Victoria Aramă, Viorica Poghirc, Cristina Popescu, Ruxandra Moroti

**Affiliations:** 1 National Institute for Infectious Diseases "Prof. Dr. Matei Balş", Bucharest, Romania; 2 Carol Davila University of Medicine and Pharmacy, Bucharest, Romania

## Background

Although the immune recovery associated with highly active antiretroviral therapy (HAART) in HIV infection has important clinical benefits, this restoration of immunity may result in deterioration, when HAART is initiated in patients with tuberculosis (TB). The immune reconstitution inflammatory syndrome (IRIS) has also been reported in non-HIV persons following anti-TB treatment (ATT). The incidence of IRIS in case-control studies on HIV and non-HIV-infected patients, has been found to be 28-36% and 7-10%, respectively, depending on background tuberculosis prevalence rates.

We report two cases illustrating IRIS in treated CNS TB, one in a patient diagnosed with HIV stage C3 and the second in a patient with immunosuppression due to anti-tumor necrosis factor alpha treatment.

## Case report

A 40 year-patient, HIV-infected, was diagnosed with tuberculous meningoencephalitis (CSF with 210 elements, lymphocytic predominance, protein of 2.1 g/L, glucose of 0.26 g/L; right hemiplegia and motor aphasia), due to *Mycobacterium tuberculosis* susceptible to all anti-TB agents. He initially improved under ATT, but one week after antiretroviral therapy (ART) was started, at 6 weeks after the initiation of ATT, he presented with worsening of his symptoms (left hemiparesis and mixed aphasia), of CSF and MRI changes. He improved after he was started on corticosteroids (dexamethasone 24 mg/day initially, then tapered doses), but was readmitted with recurrence of the left hemiparesis and worsening aphasia, while reducing the steroid dose to 8 mg of methylprednisolone. Worsening of his neurological status has been reemerging each time we try to stop steroids over a 6-month period.

The second case is a 60 year-old patient, with ankylosing spondylitis, treated for 3 years with infliximab, diagnosed with disseminated TB (multiple disseminated tuberculomas and pulmonary TB) histological and bacteriological confirmed. The initiation of ATT has led to neurological improvement, but after 3 weeks of therapy the patient presented with fever and diplopia. These symptoms improved only after corticosteroids administration (dexamethasone 16 mg/day initially, then tapered doses). At week 18 of ATT the patient was still on steroids.

## Conclusion

High doses of steroids are usually used to control the IRIS symptoms in TB patients with CNS involvement, but the dosing and duration of corticosteroids should be personalized to each patient. Some patients may require extended courses of corticosteroids.

